# SGLT2 inhibitors: a novel choice for the combination therapy in diabetic kidney disease

**DOI:** 10.1186/s12933-017-0547-1

**Published:** 2017-05-16

**Authors:** Honghong Zou, Baoqin Zhou, Gaosi Xu

**Affiliations:** 1 Medical Center of the Graduate School, Nanchang University, Nanchang, People’s Republic of China; 2 Department of Nephrology, The Second Affiliated Hospital of Nanchang University, 1, Minde Road, Donghu District, Nanchang, Zip Code: 330006 People’s Republic of China

**Keywords:** Diabetic kidney disease, SGLT-2 inhibitors, ACEI/ARBs, DPP-4 inhibitors, GLP-1 receptor agonists

## Abstract

Diabetic kidney disease (DKD) is the most common cause of end stage renal disease. The comprehensive management of DKD depends on combined target-therapies for hyperglycemia, hypertension, albuminuria, and hyperlipaemia, etc. Sodium–glucose co-transporter 2 (SGLT2) inhibitors, the most recently developed oral hypoglycemic agents acted on renal proximal tubules, suppress glucose reabsorption and increase urinary glucose excretion. Besides improvements in glycemic control, they presented excellent performances in direct renoprotective effects and the cardiovascular (CV) safety by decreasing albuminuria and the independent CV risk factors such as body weight and blood pressure, etc. Simultaneous use of SGLT-2 inhibitors and renin–angiotensin–aldosterone system (RAAS) blockers are novel strategies to slow the progression of DKD via reducing inflammatory and fibrotic markers induced by hyperglycaemia more than either drug alone. The available population and animal based studies have described SGLT2 inhibitors plus RAAS blockers. The present review was to systematically review the potential renal benefits of SGLT2 inhibitors combined with dipeptidyl peptidase-4 inhibitors, glucagon-like peptide-1 receptor agonists, mineralocorticoid receptor antagonists, and especially the angiotensin-converting enzyme inhibitors/angiotensin receptor blockers.

## Background

There are more than 350 million people suffering from type 2 diabetes mellitus (T2DM) worldwide, and its prevalence is increasing [1]. One of the most common complications in T2DM is diabetic kidney disease (DKD), which is characterized by prior diabetes mellitus, kidney damage, decreased glomerular filtration rate (GFR), and persistent albuminuria. Etiologies of DKD include environmental insults, genetic susceptibility, primarily metabolic and hemodynamic factors, etc. [2]. Thus the treatment for DKD is mainly aimed at controlling metabolic and hemodynamic abnormalities. The agents for treatment including the use of traditional anti-hyperglycemic agents (AHAs) such as metformin, insulin or pioglitazone, and renin–angiotensin–aldosterone system (RRAS) inhibitors like angiotensin-converting enzyme inhibitors (ACEI)/angiotensin receptor blockers (ARBs).

Despite a large armamentarium already being available for the management of hyperglycemia in T2DM, current oral AHAs often do not provide adequately effective or continuing glycemic control with improved β-cell function. Although metformin provides modest weight loss, most AHAs may result in weight gain or may not substantively decrease body weight [1, 3]. And renal insufficiency is definitely correlated with body fat in patients with coronary artery disease [4]. Therefore, it is necessary to call for newer AHAs that can provide long-term glycemic control and additional benefits like weight loss and the renoprotection, etc.

Sodium–glucose co-transporter 2 (SGLT2) inhibitors are new AHAs with an original insulin-independent mode of action and well tolerance, as well as favorable safety profile in patients with DKD [5, 6]. The present review provided an overview of studies with SGLT2 inhibitors, and discussed the synergistic mechanisms of SGLT2 inhibitors combined with ACEI/ARBs, dipeptidyl peptidase-4 (DPP-4) inhibitors, glucagon-like peptide-1 receptor agonists (GLP1-RAs) and mineralocorticoid receptor antagonists (MRAs) in the treatment for DKD.

## The role of SGLT2 in glucose metabolism

**Fig. 1:**
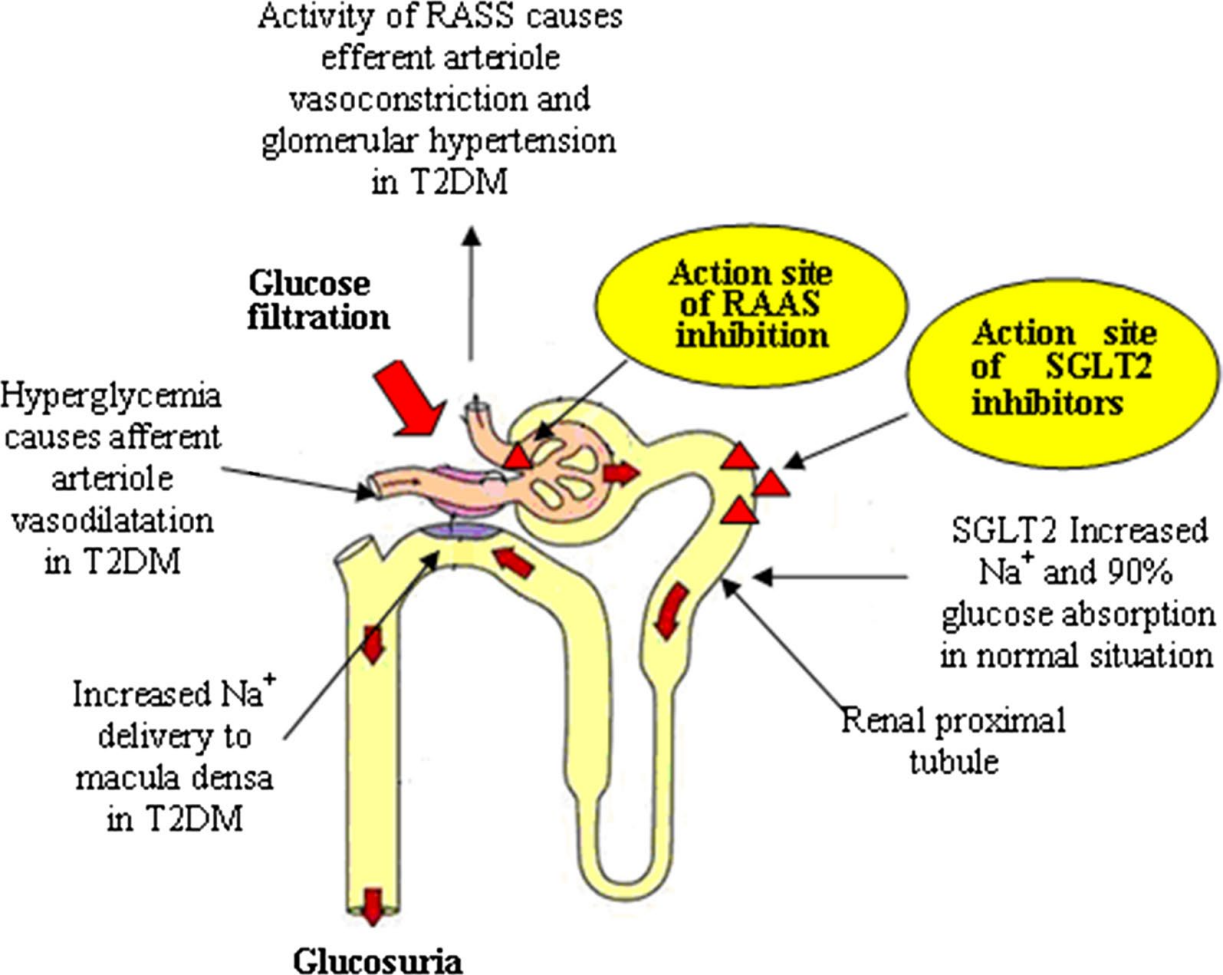
The action sites of SGLT2 and RASS inhibitors and the potential synergistic mechanism of their combined therapy in T2DM. *RASS* renin–angiotensin-aldosterone system, *T2DM* type 2 diabetes mellitus, *SGLT2* Sodium–glucose co-transporter 2

The kidneys play a critical role in the management of glucose reabsorption and maintaining the overall metabolic balance in humans [7]. The SGLT2, a low-affinity, high-capacity glucose transporter, is located primarily in the brush border membrane of the S1 segment of the proximal renal tubule and is responsible for approximated 90% of the plasma glucose reabsorption in the kidney [8]. The action site of SGLT2 in normal situation showed in Fig. 1.

Expression and activity of the SGLT2 transporter genes are up-regulated and the renal threshold is increased in patients with T2DM. These lead to increased glucose reabsorption from glomerular filtrate and reduced urinary glucose excretion (UGE), and further worsen the hyperglycemic condition [7, 9]. SGLT2 inhibitors are specifically aimed to block the reabsorption of filtered glucose in the proximal renal tubule, and resulting in increased UGE and decreased glycated haemoglobin (HbA1c) and fasting plasma glucose (FPG), especially when hyperglycaemia is present, in the meantime, they are protecting kidney [10, 11].

However, SGLT2 inhibitors could lead to a substantial increase in endogenous (hepatic) glucose production (EGP, HGP) and was accompanied by an increase in FPG concentration [12]. An acute decline in blood glucose concentration could stimulate the release of glucagon and other counter-regulatory hormones [13]. Moreover, because of the removal of the inhibitory effect of hyperglycemia on HGP, a decrease in FPG concentration potentially could result in an increase in HGP [14, 15]. Glucagon was a powerful stimulator of HGP [14–16], so the increasing glucagon observed with SGLT2 inhibitors probably provided an obvious explanation for the increase in EGP.

## The pharmacological roles of SGLT2 inhibitors in experimental models

Blocking the activity of SGLT2 leads to amelioration of renin–angiotensin system (RAS) component activation, renal inflammation and decreased expressions of antioxidant enzymes in Otsuka Long-Evans Tokushima Fatty (OLETF) rats [17]. Hence, they are slowing the progression of DKD. Furthermore, enhanced reabsorption reduces the Na–Cl–K concentration at the macula densa and increases GFR through the physiology of tubuloglomerular feedback and a possible reduction in the hydrostatic pressure in Bowman space [18]. SGLT2 inhibitors reduce hyperfiltration through the above mentioned mechanism, and attenuate/prevent the molecular markers of kidney growth, fibrotic responses of proximal tubular cells and glomerular size, as well as gluconeogenesis in diabetic Akita rats [19, 20].

For example, empagliflozin reduced the expression of nuclear deoxyribonucleic acid binding for nuclear factor kappa B (NF-κB), activator protein 1, Toll-like receptor-4 and attenuated collagen IV expression as well as interleukin-6 secretion [21]. Dapagliflozin reduced renal expression of Bax, renal tubule injury and TUNEL-positive cells and increased renal expression of hypoxia-inducible factor 1 to protect kidney [22].

## SGLT2 inhibitors in clinical trials

Currently, SGLT2 inhibitors like canagliflozin, dapagliflozin and empagliflozin have now been approved for clinical use in patients with T2DM in the United States, Europe and other countries [23]. As new AHAs, SGLT2 inhibitors have renoprotection including the following two aspects.

On one hand, SGLT2 inhibitors exert indirect renoprotection through suppressing renal glucose reabsorption to reduce blood glucose and body weight. One the other hand, SGLT2 inhibitors specifically alter renal hemodynamics and then reduce intraglomerular pressure [21, 24–26], and attenuate diabetes-associated hyperfiltration and tubular hypertrophy, as well as reduce the tubular toxicity of glucose to directly protect kidney [27]. Moreover, SGLT2 inhibitors reduce albuminuria, serum uric acid without potassium abnormalities [28], as well as BP especially systolic blood pressure (SBP) by mild natriuresis, afferent arteriole vasoconstriction, osmotic diuresis and weight loss [29]. The last but not least, diuresis can induce the increasing of hematocrit and erythropoietin. SGLT2 inhibitors reduce the workload of the proximal tubules to improve tubulointerstitial hypoxia, and then allow fibroblasts to resume normal erythropoietin production, and thereby protect the kidney [30]. Above all, SGLT2 inhibitors can be expected to translate into improved long-term kidney outcomes in patients with DKD.

A study of stage 3 DKD patients showed that canagliflozin 100 and 300 mg were associated with greater decreases in urine albumin–creatinine ratio (UACR) compared with placebo [31]. In addition, Yale et al. [31] found that the increased blood urea nitrogen (BUN) with canagliflozin occurred early and then had tendency towards baseline over the remaining treatment period. Two studies performed in T2DM patients with moderate renal impairment demonstrated that treatment with SGLT2 inhibitors led to an initial fall in estimated glomerular filtration rate (eGFR) with a trend toward an increase over time [28, 31].

Actually, sodium delivery to the macula densa could be increased by SGLT2 inhibitors, the increased sodium delivery is sensed as an increase in circulating volume at the level of the juxtaglomerular apparatus, resulting in a constriction of afferent renal arterioles, a decrease in intraglomerular pressure and a reversible decrease in single nephron GFR [18, 32]. These above mentioned investigations suggested that initial changes in renal function were associated with haemodynamic responses to SGLT2 inhibitors treatment.

The markers such as body weight (including abdominal adiposity), BP, reduced eGFR, poor control of diabetes and serum uric acid are considered as independent CV risk factors, effective management of CV risk factors and careful monitoring of eGFR may represent opportunities to reduce the risks of CV events [33]. Moreover, the reduction in CV mortality is primarily through enhancing diuresis and reducing BP [34]. SGLT2 inhibitors can efficiently normalize the above mentioned factors. The EMPA-REG OUTCOME trial and the LEADER trial have shown superiority of the SGLT2 inhibitors on the 3-point major adverse cardiovascular events (MACE) outcome and CV, as well as all-cause mortality [35].

In another several EMPA-REG OUTCOME trials, patients with T2DM at high risk for CV events who used SGLT2 inhibitors against placebo had a lower rate of the primary composite CV outcome, reduced heart failure (HF) hospitalization and CV death. Moreover, they were associated with lower rates of clinical renal events and slower progression of kidney disease. There exist consistent benefits in patients with and without baseline HF when the study drugs were added to standard care [36–39]. Although potentially atherogenic low density lipoprotein-cholesterol (LDL-C) concentrations increased by approximately 5%, this might be counterbalanced by corresponding increases in high density lipoprotein-cholesterol (HDL-C) and more significant decreases in triglyceride levels [40].

## Pharmacological synergistic effects of SGLT2 inhibitors in DKD therapy

Monotherapy with metformin, insulin or pioglitazone alone is poorly effective in maintaining long-term glycemic control and renoprotection in a majority of patients with DKD [41]. Various pharmacological approaches may be added to them as dual therapies or combined together as triple therapies. The concept of combining SGLT2 inhibitors with ACEI/ARBs, DPP-4 inhibitors and GLP1-RAs has got much attention [42]. Because the mechanisms of they complement SGLT2 inhibitors may include two aspects. On one hand, they are counteracting the SGLT2 inhibitors associated rise in EGP and glucagon, on the other hand, they are potentially applying an additive or synergistic effect on RAAS blocked and glucose-lowering to delay the progression of DKD [17].

### SGLT2 inhibitors combined with ACEI/ARBs therapy

In view of T2DM and RAS activation, it results in renal and systemic vascular dysfunction that promotes end-organ injury and significant morbidity. Guidelines recommend that inhibition of the RAS with ACEI/ARBs constitutes the therapeutic mainstay in DKD patients with albuminuria and glomerular diseases at present. It had been reported that ACEI/ARBs were associated with a lower incidence of the progression to end stage renal disease in DKD patients and reduced the incidence of composite MACE outcome [43].

The systemic RAAS would activated by natriuresis paralleled which was caused by volume depletion. Classical RAAS cascade gives rise to the production of angiotensin II (Ang II) which binds to the Ang II type 1 receptor leads to vasoconstriction, cell proliferation, inflammation, increased oxidative stress and cell apoptosis [44]. SGLT2 inhibitors would contribute to increased sodium levels delivered to the macula densa and secondary autoregulatory vasoconstriction of afferent glomerular arteriolae to neutralize the vascular imbalance driven by local Ang II characterized by glomerular hypertension in T2DM [45].

**Fig. 2:**
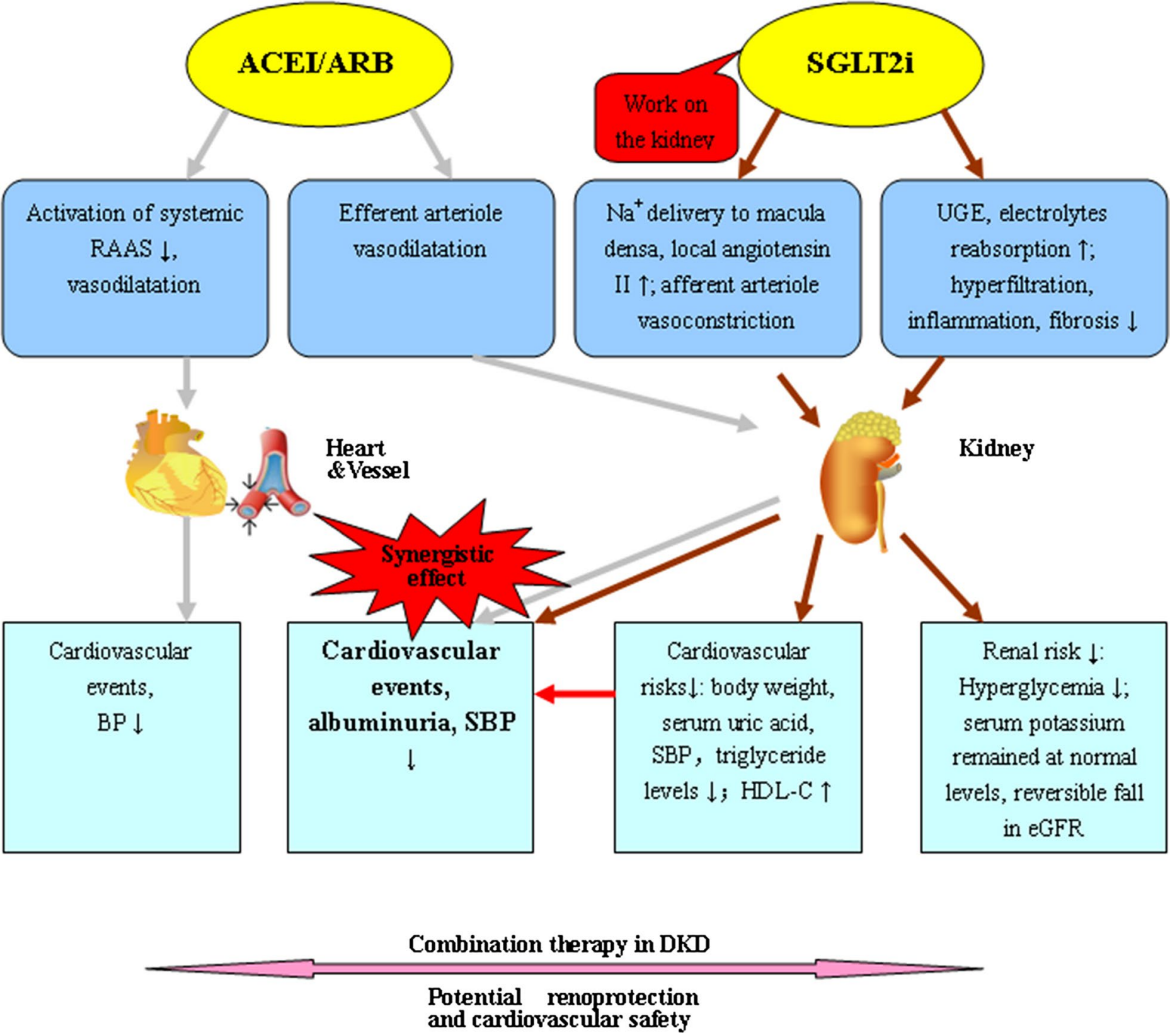
Illustration of potential synergistic effects of SGLT2 inhibitor with ACEI/ARB in DKD therapy. *ACEI*/*ARB* angiotensin-converting enzyme inhibitor/angiotensin receptor blocker, *SGLT2i* Sodium–glucose co-transporter 2 inhibitor, *RAAS* renin–angiotensin–aldosterone system, *UGE* urinary glucose excretion, *BP* blood pressure, *SBP* systolic blood pressure, *HDL*-*C* high density lipoprotein-cholesterol, *eGFR* estimated glomerular filtration rate, *DKD* diabetic kidney disease

SGLT2 inhibitors and ACEI/ARBs clearly play different roles in different places on the kidney. Figure 1 shows the action sites of SGLT2 and RASS inhibitors and the potential synergistic mechanism of their combined therapy in T2DM. Figure 2 shows the synergistic effects of SGLT2 inhibitors and ACEI/ARBs in DKD therapy.

In an experimental model study, Bautista et al. [46] found that a higher expression of SGLT2 in hypertensive rats than in normotensive rats. And the levels of protein and mRNA were decreased in rats treated with either ramipril or losartan. Therefore, they suggested that in renovascular hypertension, Ang II induced SGLT2 via the Ang II type 1 receptor possibly contributing to increased absorption of Na and thereby to the development or maintenance of hypertension.

Although SGLT2 inhibitors or ACEI/ARBs have effects on albuminuria- and BP-lowering in patients with DKD, the effects of monotherapy are always unsatisfactory. Considering their complementary mechanisms on the kidneys, theoretically, SGLT2 inhibitors and ACEI/ARBs should have synergistic action. Recently several studies indicated that the combination of SGLT2 inhibitors with ACEI/ARBs satisfactorily afforded greater renoprotection than administration of either drug alone. These results demonstrated a long-term control of hyperglycemia and BP, reduction of hyperfiltration and proteinuria, and attenuation of the development of renal injury in Dahl-STZ rat model and patients of DKD [46–48].

In a phase III clinical trial, ACEI/ARBs added-on to dapagliflozin resulted in the albuminuria and SBP decreased by −33.2% [95% confidence interval (CI) −45.4, −18.2] and −3.5 mmHg (95% CI −5.9, −1.0) compared with placebo respectively in patients with DKD. Patients had corresponding changes in HbA1c and body weight. Furthermore, trial suggested that reduction of albuminuria was to a large extent independent of glucose-lowering effects, and some other mechanisms were also involved in albuminuria-lowering effect of dapagliflozin [49]. Moreover, serum uric acid was decreased and serum potassium levels remained at normal levels by the patients in SGLT2 inhibitors treatment groups during double-blind treatment [50–52]. Table 1 reveals the benefits of SGLT2 inhibitors combined with ACEI/ARBs.

**Table 1:** Effect of SGLT2 inhibitors combined with ACEI/ARBs

Study	Participants	Intervention	Duration (weeks)	Difference amongst groups (95% CI)							
				△HbA1c (%)	△FPG (mmol/L)	△SBP (mmHg)	△Body weight (kg)	△eGFR (mL/min/1.73 m^2^)	△UACR	△Serum uric acid (μmol/L)	△Albuminuria
Heerspink [49]	A: n = 167 B: n = 189	A: DAPA 10 mg + ACEI/ARB B: ACEI/ARB + PBO	12	A–B: −0.5% (−0.7, −0.3)	NR	A–B: −3.5 (−5.9, −1.0)	A − B: −0.76 (−1.27, −0.26)	A–B: −2.80 (−5.43, −0.16)	A–B: −23.5% (−37.6, −6.3)	NR	A–B: −33.2% (−45.4, −18.2)
Weber [50]	A: n = 302 B: n = 311	A: DAPA 10 mg + ACEI/ARB B: ACEI/ARB + PBO	12	A–B: −0.49 (−0.59, −0.33)	A–B: (−0.69 vs 0.38)	A–B: −3.1 (−4.9, −1.2)	A–B: (−1.0 vs −0.3)	NS	NR	A–B: (−17.84 vs 5.95)	NR
Weber [51]	A: n = 225 B: n = 224	A: DAPA 10 mg + ACEI/ARB B: ACEI/ARB + PBO	12	A–B: −0.61% (−0.76, −0.46)	A–B: −1.2 (−1.7, −0.8)	A–B: −4.28 (−6.54, −2.02)	A–B: −0·85 (−1.39, −0.31)	NS	NS	A–B: −23.67 (−33.70, −13.64)	NR
Sha [52]	A: n = 18 B: n = 18	A: CANA 300 mg + ACEI/ARB + MET B: ACEI/ARB + PBO + MET	12	A–B: −0.6% (−0.9, −0.3)	A–B: −1.6 (−2.3, −0.9)	A–B: −13.3 (−21.2, −5.3) (supine SBP) A–B: −16.2 (−24.9, −7.5) (standing SBP)	A–B: −3.5 (−4.2, −2.7)	A–B: −4.0 (−8.7, 0.7)	NR	NR	NR

Data reported as placebo-adjusted difference (95% CI) or adjusted mean change from baseline (A group vs B group)

*SGLT2* Sodium–glucose co-transporter 2, *ACEI*/*ARBs* angiotensin-converting enzyme inhibitors/angiotensin receptor blockers, *95% CI* 95% confidence interval, *HbA1c* glycated haemoglobin, *FPG* fasting plasma glucose, *SBP* systolic blood pressure, *eGFR* estimated glomerular filtration rate, *UACR* urinary albumin creatinine ratio, *DNPA* dapagliflozin, *PBO* placebo, *NR* not reported/retrievable, *vs* versus, *NS* not-significant, *CANA* canagliflozin, *MET* metformin

However, other measures of volume status such as BUN, serum creatinine remained modestly increased and eGFR mildly decreased with SGLT2 inhibitors [50, 52]. Heerspink et al. [49] showed that the initial fall in eGFR was completely reversible only 1 week after SGLT2 inhibitors discontinuation and this reversibility indicated that the initial fall in eGFR did not reflect a decrease in the number of functioning nephrons. Besides improvements in glycemic control and lower in the above mentioned CV risks, combination therapy of SGLT2 inhibitors and ACEI/ARBs coupled with other potentially favorable renal effects may lead to a reduced long-term renal risk and CV event.

### SGLT2 inhibitors combined with DPP-4 inhibitors therapy

As previously mentioned, SGLT2 inhibitors could increase EGP (HGP) and plasma glucagon concentrations. However, DPP-4 inhibitors exert their hypoglycemic effects by preventing the degradation of endogenously released incretin hormones such as glucagon-like peptide-1 (GLP-1) and glucose-dependent insulinotropic polypeptide to enhance postprandial insulin secretion and suppress glucagon secretion, likewise, decrease endogenous (hepatic) glucose [53]. In addition, a study indicated that DPP-4 inhibitors could improve endothelial function and reduce renal and vascular oxidative stress, which was independent of albuminuria-lowering or improvement in glucose control, in patients with T2DM and chronic kidney disease [54].

Merovci et al. [12] indicated that AHAs that worked specifically on the kidney improved muscle insulin sensitivity. Since more than 80% of insulin-mediated glucose disposal during the euglycemic insulin clamp took place in skeletal muscle [55], these results indicated that the lowering plasma glucose concentration in T2DM significantly improved muscle insulin resistance. A characteristic of T2DM was that the progressive deterioration of β-cell functions. By promoting glucosuria and reducing hyperglycaemia, SGLT2 inhibitors dampened glucotoxicity, which indirectly resulted in an improvement of β-cell function and peripheral insulin sensitivity [12].

**Fig. 3:**
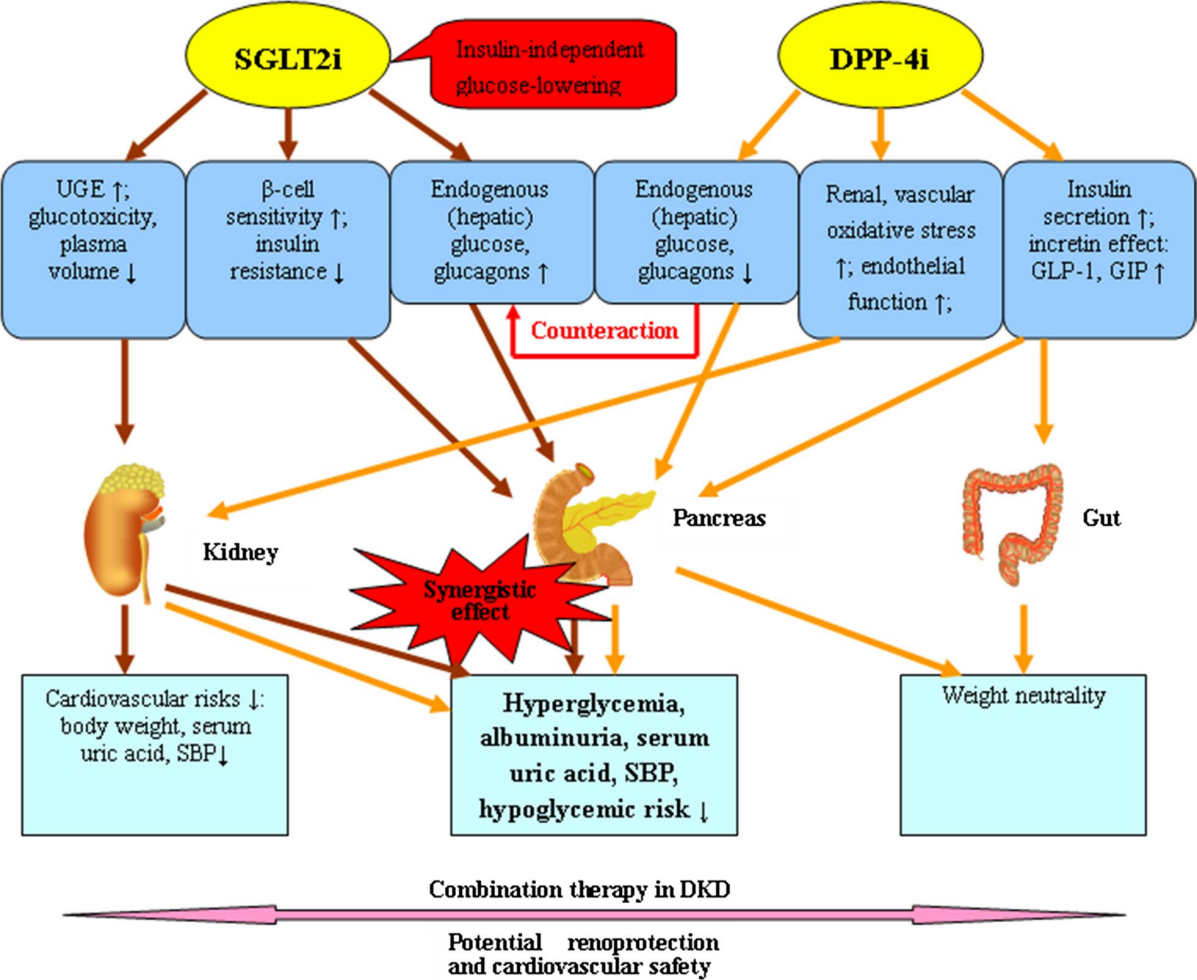
Illustration of potential synergistic effects of SGLT2 inhibitor with DPP-4 inhibitor in DKD therapy. *SGLT2i* Sodium–glucose co-transporter 2 inhibitor, *DPP*-*4i* dipeptidyl peptidase-4 inhibitor, *UGE* urinary glucose excretion, *GLP*-*1* glucagon-like peptide-1, *GIP* glucose-dependent insulinotropic polypeptide, *SBP* systolic blood pressure, *DKD* diabetic kidney disease

As mentioned above, DPP-4 inhibitor add-on to SGLT2 inhibitor meets a need for pharmacological agents with complementary mechanisms that can be applied to effectively improve glycemic control and thereby protect kidney and have potential CV safety in patients with DKD (Fig. 3).

A phase III, randomized, double-blind, parallel-group study showed that the combination of SGLT2 inhibitors and DPP-4 inhibitors resulted in the reductions of HbA1c (mean baseline 7.90–8.02% [62.8–64.1 mmol/mol]), FPG, serum uric acid, SBP, and hypoglycemic risk were superior to those with the individual components in patients with T2DM. As for, the SBP-lowering mechanisms of DPP-4 inhibitor may complement or augment SGLT2 inhibitor-induced plasma volume reduction and other mechanism. Of note, a higher proportion of subjects with microalbuminuria at baseline decreased to no albuminuria at the end of combination treatment [56]. Mean changes from baseline in UACR and eGFR were small and similar across treatment groups [57]. Furthermore, the combined therapy had CV safety and did not induce weight gain in several large prospective CV outcome studies, as well as potential renoprotection [53, 56]. Pharmacokinetics and pharmacodynamic (PK–PD) study investigated in healthy individuals found no drug–drug interaction in combination therapy of SGLT2 inhibitors and DPP-4 inhibitors [58].

### SGLT2 inhibitors combined with GLP1-RAs therapy

It had been reported that GLP1-RAs could inhibit the potent inflammatory mediator NF-κB and decrease monocyte chemoattractant protein-1, intracellular cell adhesion molecule-1 and vascular cell adhesion molecule-1, which had been associated with abnormalities in vascular function and progression of DKD [59, 60]. Moreover, GLP1-RAs could induce significant diuretic and natriuretic responses and might have beneficial effects on renal sodium and water handling [61].

**Fig. 4:**
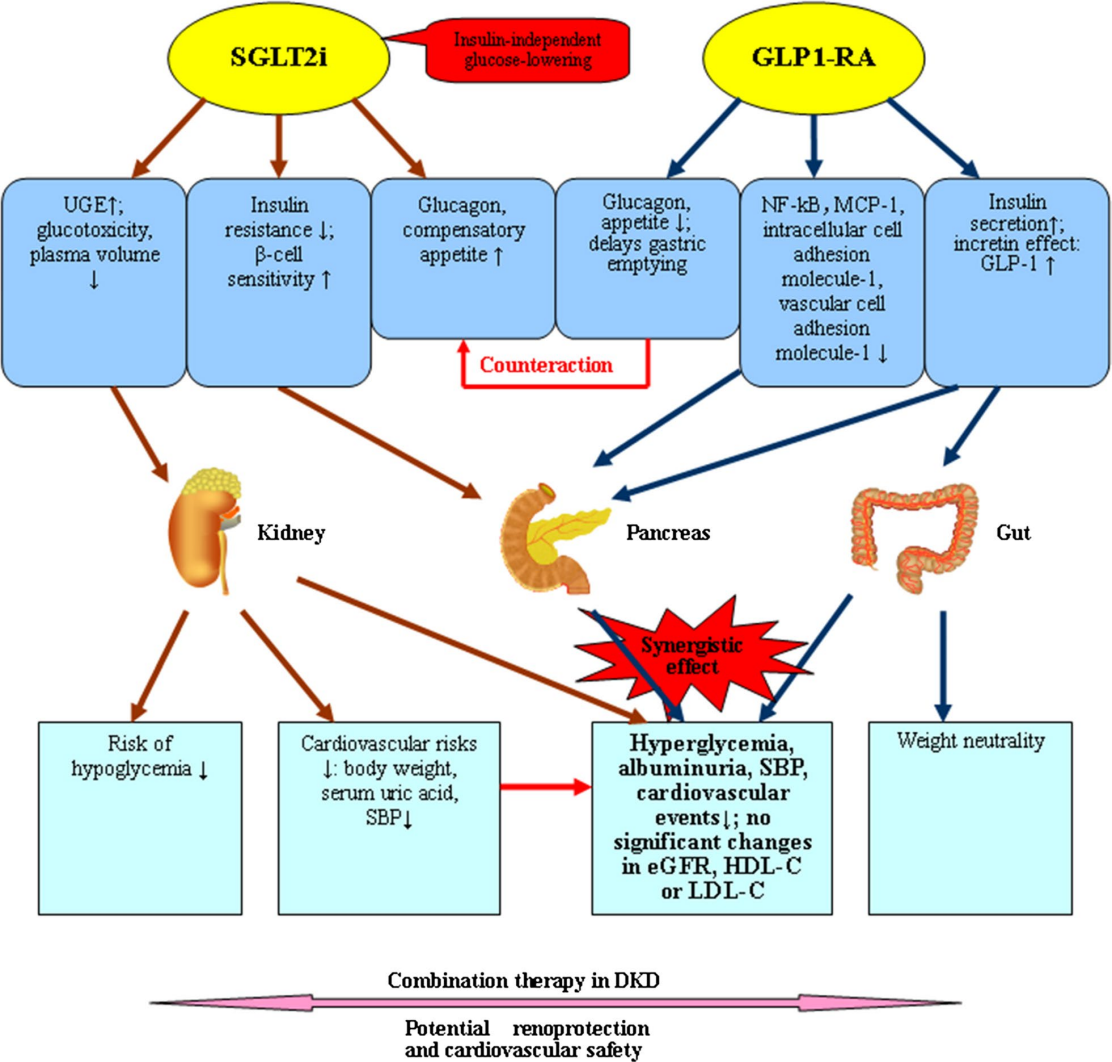
Illustration of potential synergistic effects of SGLT2 inhibitor with GLP-1 receptor agonists in DKD therapy. *SGLT2i* Sodium–glucose co-transporter 2 inhibitor, *GLP1*-*RA* glucagon-like peptide-1 receptor agonist, *UGE* urinary glucose excretion, *NF*-*κB* nuclear factor kappa B, *MCP*-*1* monocyte chemoattractant protein-1, *GLP*-*1* glucagon-like peptide-1, *SBP* systolic blood pressure, *eGFR* estimated glomerular filtration rate, *HDL*-*C* high density lipoprotein-cholesterol, *LDL*-*C* low density lipoprotein-cholesterol, *DKD* diabetic kidney disease

GLP1-RAs stimulated the GLP-1 receptor to increase insulin secretion and inhibited glucagon secretion in a glucose-dependent manner. Elevated glucagon levels induced by SGLT2 inhibitors could be counteracted by GLP1-RAs. SGLT2 inhibitors decreased calorie availability through the urine to reduce body weight, otherwise, weight loss might be attributed to visceral fat tissue lipolysis and enhanced lipid metabolism. But this calorie loss could lead to a compensatory appetite increase [24, 62]. However, GLP1-RAs lost body weight via reduced appetite and delayed gastric emptying [63]. The mechanisms of two combined drugs may have complemented each other and have potential renoprotection and CV safety (Fig. 4).

Several studies indicated that reduction of HbA1c, SBP and body weight was observably lower in patients with combination therapy than without. No significant changes in eGFR, HDLC or LDL-C were found in both groups [64, 65]. Especially, adverse effects of hypotension or dehydration in dapagliflozin/exenatide-treated participants were not reported. The study showed that combined therapy was superior to monotherapy.

### SGLT2 inhibitors combined with MRAs therapy

It has been reported that Aldosterone contributes to renal injury. MRAs have effect on renoprotection by reducing the concentration of aldosterone in DKD patients. But MRAs are limited by notable side effect hyperkalaemia. An experimental model study demonstrated that spironolactone prevented the STZ-induced increase in the renal aldosterone synthase *CYP11B2* mRNA content and did not influence glycemic level or BP in STZ-induced diabetic rats. Moreover, controlling blood glucose with AHAs also attenuated the renal expression of mRNA for *CYP11B2*. These results indicated that spironolactone exerted renoprotective effects and inhibited local angiotensin-converting enzyme expression and the hyperglycemia-induced overexpression of *CYP11B2* in the kidney [66].

The novel drug finerenone of MRAs is more selective for the mineralocorticoid receptor than spironolactone and has greater affinity for the mineralocorticoid receptor than eplerenone, and shows less incidence of hyperkalemia as compared to spironolactone. Hence, it can efficiently reduce the concentration of aldosterone and play a role in renoprotection [67]. Furthermore, a large randomized controlled trial (RCT) on the renoprotective effect of finerenone in DKD is currently ongoing (FIDELIO-DKD).

As previously mentioned, SGLT2 inhibitors played roles in lowering glucose, renoprotection and CV safety [24]. It was worth noting that SGLT2 inhibitors were not increasing the risks of hyperkalemia or severe hypokalemia [28, 68]. The apparent mechanism of action in SGLT2 inhibitors and MRAs suggests that there exists a potential synergistic renoprotective effect of combination therapy to slow the progression of DKD. Large RCTs are imperative to investigate if this combination therapy can provide effective renoprotection in DKD.

## The safety of combined therapy with SGLT2 inhibitors

Although the combined therapy have potential renoprotection and CV safety, receiving SGLT2 inhibitors may lead to adverse effects like genital mycotic and urinary tract infections even it combined with other drugs [29, 69]. This may be attributed to the increased and prolonged glucosuria induced by SGLT2 inhibitors. And then, adverse effect of SGLT2 inhibitors therapy reported by a study is osmotic diuresis which leads to dehydration, hypotension and renal impairment. Intravascular volume depletion, a dose-dependent increase in serum creatinine and a known marker of renal damage are also discovered in SGLT2 inhibitors treatment in patients with T2DM [70]. In addition, efficacy in lowering glucose levels of SGLT2 inhibitors depends in the filtration rate in the kidneys, so it has shown no efficacy in reducing HbA1c levels in patients with severe renal impairment, ESRD and patients on dialysis, as well as some moderate renal impairment [28].

Above all, although these side effects are in most cases not of serious nature, patients and the health care providers should be aware of them, in any case. In addition, SGLT2 inhibitors should be prescribed with caution in T2DM patients with significant renal impairment. Maybe health care providers should limit the capacity of this drug class in treating severe DKD patients.

## Conclusions

All the researches, mentioned above, demonstrating the SGLT2 inhibitors with their novel mechanism and associated benefits on glucose-lowering, renoprotection, body weight, CV safety, etc. have proved to be promising choices either as monotherapy or as combination therapy for patients with DKD. Moreover, large RCTs are necessary to investigate the combination therapy of SGLT2 inhibitors with the above mentioned drugs and thereby provide the direct evidences of renoprotection and CV safety in DKD patients.

## Abbreviations

ACEI: angiotensin-converting enzyme inhibitors
AHAs: anti-hyperglycemic agents
Ang II: angiotensin II
ARBs: angiotensin receptor blockers
BP: blood pressure
BUN: blood urea nitrogen
CI: confidence interval
CV: cardiovascular
DKD: diabetic kidney disease
DPP-4: dipeptidyl peptidase-4
eGFR: estimated glomerular filtration rate
EGP: endogenous glucose production
FPG: fasting plasma glucose
GFR: glomerular filtration rate
GLP-1: glucagon-like peptide-1
GLP1-RAs: glucagon-like peptide-1 receptor agonists
HbA1c: glycated haemoglobin
HDL-C: high density lipoprotein-cholesterol
HF: heart failure
HGP: hepatic glucose production
LDL-C: low density lipoprotein-cholesterol
MACE: major adverse cardiovascular events
MRAs: mineralocorticoid receptor antagonists
NF-kB: nuclear factor kappa B
OLETF: Otsuka Long-Evans Tokushima Fatty
PK-PD: pharmokinetics and pharmacodynamic
RAAS: renin–angiotensin–aldosterone system
RAS: renin–angiotensin system
RCT: randomized controlled trial
SBP: systolic blood pressure
SGLT2: sodium–glucose co-transporter 2
STZ: streptozocin
T2DM: type 2 diabetes mellitus
UACR: urine albumin–creatinine ratio
UGE: urinary glucose excretion
