# Correction to: SGLT2 inhibitors: a novel choice for the combination therapy in diabetic kidney disease

**DOI:** 10.1186/s12933-018-0676-1

**Published:** 2018-03-09

**Authors:** Honghong Zou, Baoqin Zhou, Gaosi Xu

**Affiliations:** Department of Nephrology, The Second Affiliated Hospital of Nanchang University, No. 1, Minde Road, Donghu District, Nanchang, Zip Code: 330006 People’s Republic of China

## Correction to: Cardiovasc Diabetol (2017) 16:65 10.1186/s12933-017-0547-1

Following publication of the original article [1] the authors reported that the first affiliation (“Medical Center of the Graduate School, Nanchang University, China”) had been added in error, and that the correct author information is as given in this erratum.
